# Parameter-Efficient Adaptation of Large Vision—Language Models for Video Memorability Prediction

**DOI:** 10.3390/s25061661

**Published:** 2025-03-07

**Authors:** Iván Martín-Fernández, Sergio Esteban-Romero, Fernando Fernández-Martínez, Manuel Gil-Martín

**Affiliations:** Grupo de Tecnología del Habla y Aprendizaje Automático (THAU Group), Information Processing and Telecommunications Center, E.T.S.I. de Telecomunicación, Universidad Politécnica de Madrid (UPM), 28040 Madrid, Spain; sergio.estebanro@upm.es (S.E.-R.); fernando.fernandezm@upm.es (F.F.-M.); manuel.gilmartin@upm.es (M.G.-M.)

**Keywords:** large visual language models, video memorability, multimedia perception, efficient adaptation

## Abstract

The accurate modelling of video memorability, or the intrinsic properties that render a piece of audiovisual content more likely to be remembered, will facilitate the development of automatic systems that are more efficient in retrieving, classifying and generating impactful media. Recent studies have indicated a strong correlation between the visual semantics of video and its memorability. This underscores the importance of developing advanced visual comprehension abilities to enhance model performance. It has been demonstrated that Large Vision–Language Models (LVLMs) demonstrate exceptional proficiency in generalist, high-level semantic comprehension of images and video, due to their extensive multimodal pre-training on a vast scale. This work makes use of the vast generalist knowledge of LVLMs and explores efficient adaptation techniques with a view to utilising them as memorability predictors. In particular, the Quantized Low-Rank Adaptation (QLoRA) technique is employed to fine-tune the Qwen-VL model with memorability-related data extracted from the Memento10k dataset. In light of existing research, we propose a particular methodology that transforms Qwen-VL from a language model to a memorability score regressor. Furthermore, we consider the influence of selecting appropriate LoRA hyperparameters, a design aspect that has been insufficiently studied. We validate the LoRA rank and alpha hyperparameters using 5-Fold Cross-Validation and evaluate our best configuration on the official testing portion of the Memento10k dataset, obtaining a state-of-the-art Spearman Rank Correlation Coefficient (SRCC) of 0.744. Consequently, this work represents a significant advancement in modelling video memorability through high-level semantic understanding.

## 1. Introduction

The overwhelming abundance of multimedia content presents a significant challenge in identifying which pieces will leave a lasting impression on audiences. Effective audience engagement is crucial in various sectors, including cinema, television, advertising, education, and Internet content development. Consequently, there is a critical need for systems capable of ranking, classifying, and retrieving media items based on their relevance to viewers. In this context, the concept of memorability emerges as a valuable framework for understanding and predicting the impact of multimedia content. Being able to predict how memorable a piece of audiovisual content is can help build systems that recommend, filter out, or even modify pieces of media based on this variable. Currently, systems based on predicting media memorability are being used in the marketing field to aid in predicting the impact of marketing campaigns [1,2,3]. These uses can be extended to other disciplines, such as helping educators to create more memorable support materials.

Memorability is a concept rooted in human perception and is studied in the disciplines of philosophy, psychology, and neuroscience. Perception involves gathering, organizing, and processing information from the environment and is essential in various contexts, including object recognition [4] and aesthetic experiences [5]. It relies on the ability of the sensory system to use multimodal signals—concurrent stimuli in multiple sensory modalities—and is influenced by cognitive functions such as expectancy, learning, and attention [4]. Memory, a crucial cognitive function, encodes, stores, and retrieves perceived information [6]. Identifying the features of stimuli that enhance their retention in memory allows us to understand the basic mechanisms of the perceptual system and to develop artificial systems with similar capabilities.

Memorability can be studied as the innate quality of an image or video, determined by its audiovisual characteristics, that influences how easily it can be recalled in the future [7]. Perception is significantly affected by this feature of visual stimuli. Although memory may seem like a subjective cognitive process, research in psychology and neuroscience suggests that certain visual elements are inherently more memorable [8,9,10]. Studies have found a strong correlation between the contextual and semantic qualities of an image and its recall value [11]. Furthermore, the analysis of image semantics involves not only visual data, but also textual methods, such as descriptions of natural language [12], which highlight the multifaceted nature of memorability.

The intrinsic relationship between semantics and memorability motivates the use of tools and models that possess advanced analytical and reasoning capabilities. This could enable a deeper understanding of the complex aspects of multimedia content and how they relate to human perception. Multi-Modal Large Language Models (MM-LLMs) and Large Vision–Language Models (LVLMs) have proven themselves as the next frontier in automatic reasoning and content understanding, showcasing outstanding performance in several intricate tasks such as Visual Question–Answering [13,14], Image Captioning [15,16], and Refer Expression Comprehension [17]. Moreover, they have become powerful companion tools for instruction-based tasks, and their use as a commercial product is continuously expanding. This paper aims to leverage and adapt the advanced knowledge that LLMs present in order to generate more informed and precise memorability estimators of multimedia content. Specifically, we introduce a series of modifications performed on the original LVLMs, which are trained to generate textual output, such as descriptions or conversations, in order to turn them into memorability score regression models. We also report state-of-the-art performance in predicting memorability scores on the Memento10k corpus test set [18] with a Spearman Rank Correlation Coefficient (SRCC) of 0.744 by applying these techniques to the Qwen-VL model [19]. Our main contributions include the following:

1. Introducing the label pre-processing and prediction post-processing necessary for using LVLMs for regression.
2. Exploring Parameter-Efficient Fine-Tuning (PEFT) of LVLMs, and more concretely Quantized Low-Rank Adaptation (QLoRA) [20] of LVLMs for memorability prediction.
3. Evaluating our proposal on the Memento10k dataset, obtaining state-of-the-art performance.

The remainder of this paper is structured as follows. Section 2 provides an overview of related work, including traditional approaches to computational memorability prediction, the evolution and applications of Large Vision–Language Models (LVLMs), and their use in memorability prediction. Section 3 outlines the materials and methods used, detailing our proposal, the dataset used, and the experimental setup. Section 4 presents the results and discussion, covering the outcomes of zero-shot inference, an exploration of LoRA hyperparameters, error analysis, and a comparison against state-of-the-art methods. Finally, Section 5 concludes the paper with a summary of the findings and suggestions for future work.

## 2. Related Work

### 2.1. Traditional Approaches to Computational Memorability Prediction

The processes involved in the acquisition, storage, and retrieval of visual information are not unfamiliar to psychologists and neuroscientists. Under appropriate conditions, humans tend to show an almost limitless ability to correctly recognize stimuli that are presented to them [21]. Human memory capacity has been shown to be higher for pictorial material than for other sources such as verbal [22]. Researchers have even identified activations in different areas of the brain responsible for image and object encoding, as well as their relationship with image memorability [23,24]. Interestingly, there is evidence that certain types of stimuli are inherently easier to remember than others, despite the subjective preferences and biases of the individuals under study [8,9,10]. Based on this, a line of research arises that tries to analyze this intrinsic characteristic of multimedia and create computational systems that try to predict memorability based on them.

In the realm of still images, seminal work by Isola et al. [7] showed that, in the absence of familiar components, certain objects and scenes tend to be more memorable than others. This pinpoints the importance of object understanding and scene parsing when predicting memorability. Perera et al. [25] built on these observations to build a system that matches human performance in the LaMem dataset [26].

A natural extension from image to video arises thanks to studies that are carried out mainly using the Memento10k [18] and VideoMem datasets [27], with special mention to the MediaEval Predicting Video Memorability Challenge that each year fosters new and bold ideas that explore innovative memorability prediction systems [28,29,30]. Constantin and Ionescu  [31] trained a Video Transformer using the portion of the video that is, on average, the most remembered by the annotators, thus obtaining a top SRCC score of 0.665 on the official test partition of the Memento10k dataset.

Semantic properties have also been proven to be key in the analogous task of video memorability prediction. In particular, the use of textual information to convey semantic value to image descriptors has given profitable results [12,32,33,34]. Dumont et al. achieved superior performance by integrating low-level descriptors, scene parsing information, and event understanding together with a contextual module that took into account the similarity between each sample and the rest of the corpus [35]. Li et al. proposed an adaptive ensemble of similar modules in order to account for possible imbalances in the importance that each of them has in predicting video memorability [36]. However, in a preliminary work, Kumar et. al showed that training an architecture that simply attends to several frames in the video using a Transformer model mimics human gaze with learnt attention patterns, achieving a top SRCC score of 0.71 over the official testing set of Memento10k [37]. In conclusion, according to recent work, an accurate video memorability prediction system should combine intrinsic image properties with semantic attribute, scene, action, and object representation.

### 2.2. LVLMs and Their Applications

The advent of Large Language Models (LLMs) in recent times has shaken the field of artificial intelligence (AI), shifting towards a completely new paradigm. These new models have opened the door to discussions about machine consciousness, spontaneous emotion generation, and even Artificial General Intelligence (AGI), which has seemed beyond imaginable to date [38,39,40,41]. In general, an LLM is defined as an auto-regressive, Transformer-based [42] (encoder–decoder, encoder-only, or decoder-only) model that has been pre-trained in an unsupervised manner using a vast amount of unlabeled text, usually scraped from the Web. The pre-training task is typically Next Token Prediction (NTP), where the model tries to predict the next textual element of a sequence conditioned on the previous information:

(1) $$L=-\frac{1}{T}\sum_{t=1}^{T}\log P\left(y_{t}|y_{<t};\theta \right)$$

where

*T*     is the sequence length;

$y_{t}$    is the actual token at time *t*;

$y_{<t}$  represents all tokens before time *t*;

$P(y_{t}|y_{<t};\theta )$  is the probability of the token $y_{t}$ given the preceding tokens $y_{<t}$ and model parameters θ.

State-of-the-art Large Language Models (LLMs) have demonstrated exceptional performance in a wide range of Natural Language Processing (NLP) tasks, including machine translation, document understanding, code generation, and mathematical reasoning [43,44,45,46,47]. Although these cutting-edge models prioritize performance, they often require substantial computational resources. In contrast, more efficient LLMs, which maintain a strong subset of these capabilities, have shown remarkable adaptability and effectiveness in handling complex tasks [48,49,50].

Recent advancements in artificial intelligence have shifted from text-only Large Language Models (LLMs) to Large Vision–Language Models (LVLMs) and, more broadly, Multi-Modal LLMs (MM-LLMs). These models aim to extend their inherent general knowledge to comprehend and process additional modalities, such as images, videos, and audio, either independently or in combination.

LVLMs typically integrate a pre-trained image encoder with a “translation module”, which maps visual embeddings into the latent input space of the LLM. This adaptation process often employs multi-stage training on extensive multimodal datasets [19,51,52,53]. Leveraging their robust pre-training, advanced reasoning capabilities, and adaptability, LVLMs have demonstrated significant efficacy across a broad range of computer vision tasks [54,55]. However, a key challenge remains in refining LVLMs from general-purpose conversational agents into task-specific systems optimized for classification or regression.

### 2.3. LVLMs for Memorability Prediction

Cutting-edge approaches for memorability prediction have relied on LVLMs as part of their solution because of their aforementioned advantages. In recent preliminary studies, Singh et al. [56] reported that the instruction fine-tuning of the LLaMa-VID model with behavior content in the form of YouTube comments, views, likes, etc., improves its content-understanding abilities, including memorability prediction, with a reported top SRCC score of 0.71 over the validation set of the Memento10k corpus. SI et al. [57] proposed a system to predict long-term ad memorability based on end-to-end adaptation of a Llama LLM [50] and an EVA-CLIP image encoder [58]. They reported a top SRCC result of 0.75 over the validation set of the Memento10k corpus. Both approaches yielded promising results by leveraging the capabilities of LVLM-based architectures. However, they require training billions of parameters, which requires significant computational resources and time. In this work, we explore a method for adapting these large models to achieve comparable performance while training a reduced set of parameters, which reduces computational cost and training time. By applying the QLoRA technique to the Qwen-VL LVLM, we aim to efficiently leverage the vast knowledge present in the original system, obtained through large-scale pretraining, shifting its behavior to a memorability prediction and achieving cutting-edge performance without the computational demands required for full fine-tuning. While there exists evidence of the wide capabilities of LVLMs in complex multimodal reasoning and semantic understanding, applying these skills to a downstream task with limited resources poses a challenge that we tackle through efficient fine-tuning.

## 3. Materials and Methods

In this section, we detail our proposed solution for LVLM-based memorability prediction and the setup employed to carry out the necessary experiments. Section 3.1 will explain our proposed architecture, including the base LVLM, the prompting technique, and the adaptation module. We will introduce the data used for experimentation in Section 3.2 and the experimental setup in Section 3.3.

### 3.1. Proposal

**Figure 1:**
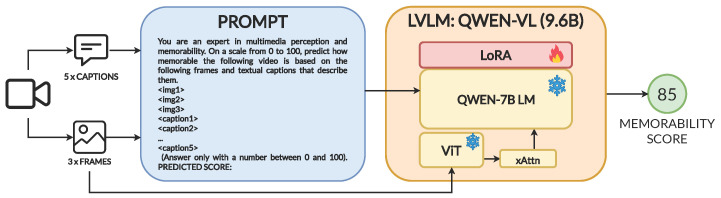
A visual representation of our proposed framework for predicting video memorability. Given three video frames and five textual captions as input, our model, powered by the Qwen-VL LVLM, estimates the likelihood of the video being remembered as a score ranging from 0 to 100. The weights of the LVLM are kept frozen, as indicated by the snowflake, and the Low-Rank Adaptation module is trained on the downstream task (flame symbol).

A schematic view of our proposal is shown in Figure 1. Our main objective is to benefit from the extensive and general pre-training knowledge displayed by LVLMs, steering their behavior from open-world language generation to memorability score prediction. We employ the Qwen-VL model [19] due to its relatively small size (9.6 billion parameters total), versatility, and robustness. Qwen-VL is built upon the Qwen-7B language model, enhanced with a visual processing capability. Its architecture comprises three key components: a visual encoder, a position-aware vision–language adapter, and an LLM as a text decoder. The visual encoder is based on a Vision Transformer (ViT), which processes up to three input images by segmenting them into patches and generating a sequence of image features. To efficiently integrate visual and textual modalities, the model incorporates a position-aware vision–language adapter, which employs a single-layer cross-attention module with learnable query embeddings. This adapter compresses the visual feature sequence to a fixed length of 256 while preserving spatial information through 2D absolute positional encodings. The transformed visual representations are then fed into the Qwen-7B LLM, which is responsible for generating responses based on both visual and textual inputs. The input–output interface utilizes special tokens to delineate different modalities, effectively enabling attentive processing between visual and textual tokens. The model undergoes a three-stage training process: large-scale pretraining on image–text pairs, multi-task fine-tuning with high-resolution images, and supervised instruction tuning for interactive vision–language understanding.

In this work, we use the Qwen-VL-Chat-Int-4 checkpoint (https://huggingface.co/Qwen/Qwen-VL-Chat-Int4 (accessed on 4 March 2025), which incorporates two main advantages over the base model. Firstly, it has undergone Supervised Fine-Tuning (SFT) adaptation, making it more versatile and flexible. Secondly, its weights have been quantized to 4-bit integers, which drastically reduces size whilst keeping most of its predictive abilities. This goes hand in hand with our goal of benefiting from large-scale models with reduced computing resources.

The prompt used to send visual and textual information about the video and task to the model is shown in Listing 1. The first two sentences are used to instruct the model and condition its generation. Then, we include three frames extracted from the video: the first, middle, and last. Given that the videos in Memento10k are short and recorded in amateur setups, they often include a single shot and semantic unit. Therefore, this frame selection strategy has been used in previous work as a compromise between the overall representation of the visual information in the scene and computational resources [32,34,59,60,61,62]. In our case, the Qwen-VL-Chat model under study accepts a maximum of three images, which justifies our choice of this method. Finally, we append the five available textual descriptions for that video and conclude the prompt with a last conditioning sentence. The model is prompted to generate an integer score between 0 and 100 to control variance originating from the generation of uncontrolled non-integer numbers, as has been performed in a previous work [57].

**Listing 1.** Prompt used to feed the Qwen-VL model with visual and textual information about the
video as well as context about the task and a call for action.

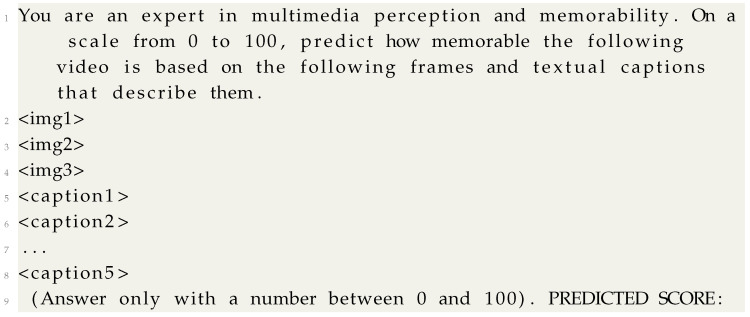



The images are encoded by a Visual Transformer (ViT) and then projected into the language embedding space via cross-attention with a set of learnable query vectors before being fed to the language model. To adapt the model for memorability prediction, we use Low-Rank Adaptation (LoRA) [20], a PEFT technique that freezes the pre-trained LVLM parameters and adds a small, trainable network to learn a linear transformation of the output. This allows efficient specialization of a large, general-purpose model, requiring significantly less computational effort than fine-tuning the full model. The adaptation process can be defined as follows:

(2) $$h=W_{0}x+\frac{\alpha }{r}\Delta Wx$$

where

*h*    is the output of the adapted network;

*x*    is the input;

$W_{0}$    is the weight matrix of the original network;

ΔW  represents the learnt LoRA weight matrix of rank *r*;

α       is a multiplicative factor that controls the importance given to the appended weights versus the original.

The hyperparameters α and *r* control the influence of the newly adapted weights. Although the authors of the original formulation set them empirically and suggest a ratio of α/r=2, recent works have shown that the choice of these values is highly problem-dependent and can have a significant impact on downstream performance [63,64]. In this case, where model weights are casted to 4-bit integers, the adaptation process is referred to as Quantized LoRA or QLoRA [65].

### 3.2. Dataset

We carry out our experiments using the Memento10k dataset [18], a collection of 10,000 videos that last 3 seconds on average. The scores are continuous: a number between 0 and 1 is associated with the ability of certain video to be remembered in general. These annotations are generated through a memory game, wherein participants watch a series of videos, which may include repetitions, and indicate by pressing a button whether they remember viewing them earlier during the game. The objective is to assess how video recall diminishes as the interval between repetitions extends in the previously mentioned memory game. Considering the brevity of the videos, it is likely that they embody a singular semantic concept.

A notable characteristic of the dataset is that the videos originated from web scraping, which generally results in reduced image quality due to recording with less expensive consumer devices. However, the emphasis is on human actions and movements, with the samples exhibiting substantial optical flow. The Memento10k dataset includes five textual descriptions per video, referred to as closed captions (CC), or simply captions. These short sentences concisely encapsulate the main attributes of each clip, emphasizing semantic content while excluding emotional elements. This characteristic favors the integration of semantic information through natural languages, thus enriching the original visual attributes.

### 3.3. Experimental Setup

Regarding our internal experiments, we evaluate the effect of QLoRA fine-tuning against zero-shot inference using the original Qwen-VL model. We also explore the impact of the LoRA hyperparameters on downstream performance. To that end, we evaluate our experiments using 5-Fold Cross-Validation using the concatenation of all labeled samples from the Memento10k corpus—training and validation sets, comprising a grand total of 8500 videos. In this way, we isolate the performance evaluation from the possible particularities of the validation set distribution. We use the mean Spearman Correlation Coefficient over the five folds as a figure of merit, in concordance with the literature. Lastly, we perform inference on the 1500 unlabeled samples from the test set that is used as part of the MediaEval Predicting Video Memorability challenge [28,29,30]. We submit our run to the challenge organizers in order to obtain a performance metric on said data. All models are trained with a batch size of 4 for 4 epochs using a single 40 GB NVIDIA A100 GPU (NVIDIA, Santa Clara, CA, USA).

## 4. Results and Discussion

### 4.1. Zero-Shot Inference

**Figure 2:**
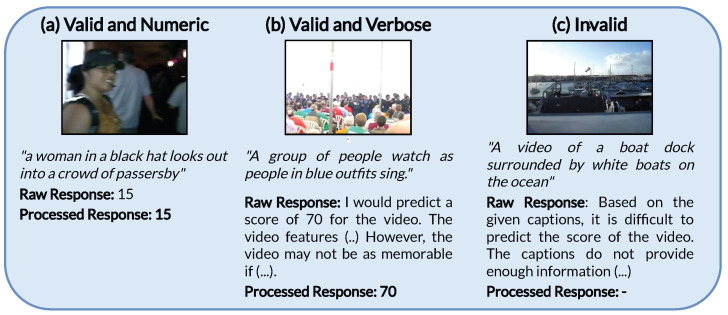
Examples of responses given by the zero-shot model when prompted about memorability scores. The answer can be just a number (**a**), a paragraph including a numerical result that can be correctly parsed (**b**), or text with no valid integer prediction (**c**).

**Figure 3:**
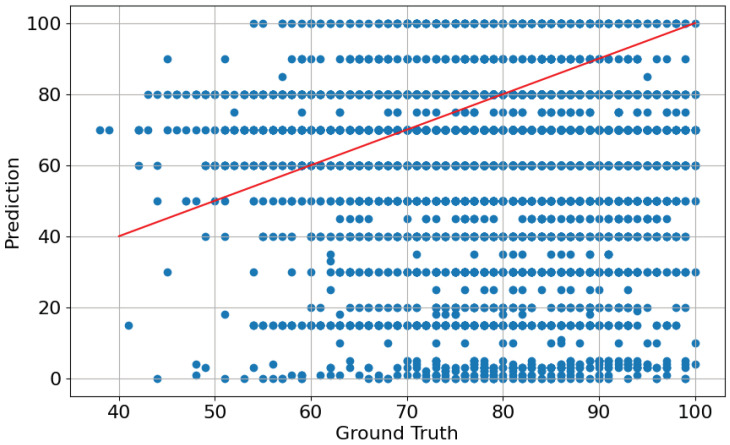
Scatter plot of the validation predictions and ground truths for the zero-shot Qwen-VL model. Each blue dot represents a video sample. The red line represents the perfect estimator: Ground Truth = Prediction.

First, we explore the zero-shot capabilities of Qwen-VL by performing inference on all labeled data from Memento10k using the prompt described in Section 3.1. As can be seen in Figure 2, when used in inference mode the LVLM might not always adjust to the desired numerical output but rather produce full sentences containing a possibly valid numerical value that must be extracted. For that reason, we post-process the results, using regular expressions to find and extract a number between 0 and 100 from the model output. If no such number is found, we consider the prediction invalid and assign the mean score of the entire training dataset (80) for evaluation. After filtering 600 invalid responses from the 8500 total (7.06%), we obtain an SRCC value of **−0.050**. This suggests that, in spite of the large amount of pre-training data and the vast knowledge acquired in the first learning stages, memorability prediction as a regression problem falls out of the scope of the zero-shot capabilities of the model. This intuition is supported by prediction points, as shown in Figure 3. The predicted values are shown to be binned to a few discrete values that show no correlation with the ground truth. Therefore, domain adaptation is necessary to guide the model in relating visual and semantic cues from the video samples effectively to their potential memorability.

### 4.2. LoRA Hyperparameter Exploration

**Figure 4:**
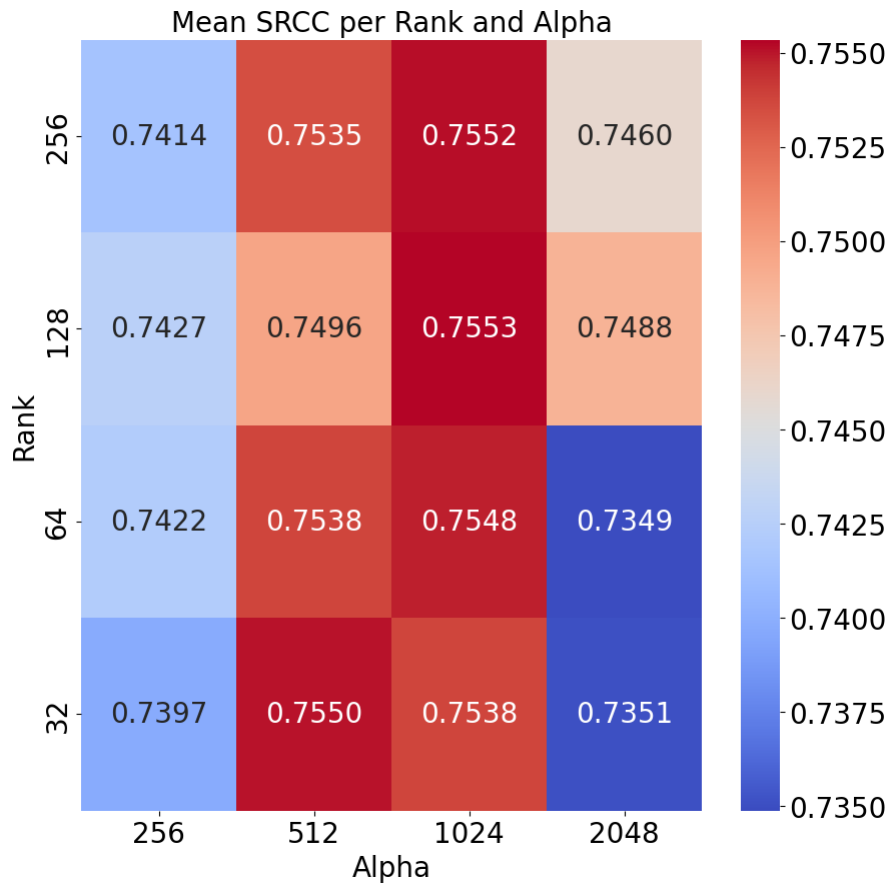
Heatmap of the mean SRCC over the five folds for each alpha-rank configuration tested.

The mean performance in terms of SRCC in the five folds for all alpha-rank configurations tested is shown in Figure 4. First, it should be noted that all results exceed 0.73, demonstrating the effectiveness of this method. By training a relatively smaller network, the pre-trained knowledge of the base LVLM can be leveraged to build a strong memorability predictor. Moreover, a top result of **0.7553** is obtained using 128 rank matrices with an alpha parameter of 1024. In general, the observed tendencies highlight the significant impact of selecting an appropriate alpha parameter. A low value suggests under-utilization of the adaptation module, therefore neglecting the acquired task-specific knowledge. Conversely, an excessively high alpha value can diminish performance by overshadowing the base inherent image and language understanding capabilities held by the model. Overall, these results demonstrate the beneficial synergy between a large general-purpose foundation model and a smaller task-specific adapter.

With respect to the *rank* parameter, it appears to have a less pronounced effect on model performance. This value represents the rank of the matrix ΔW that is learned as an adapter. Although performance variations are not substantial, achieving the best result with a rank of 128 suggests that, for this particular problem and model setup, a rank-128 decomposition most effectively characterizes the original weight matrices. Overall, this study demonstrates that the appropriate selection of LoRA hyperparameters can greatly impact the performance of the model. Although we used a grid search for this purpose, future work should explore more efficient hyperparameter optimization strategies.

### 4.3. Error Analysis

**Figure 5:**
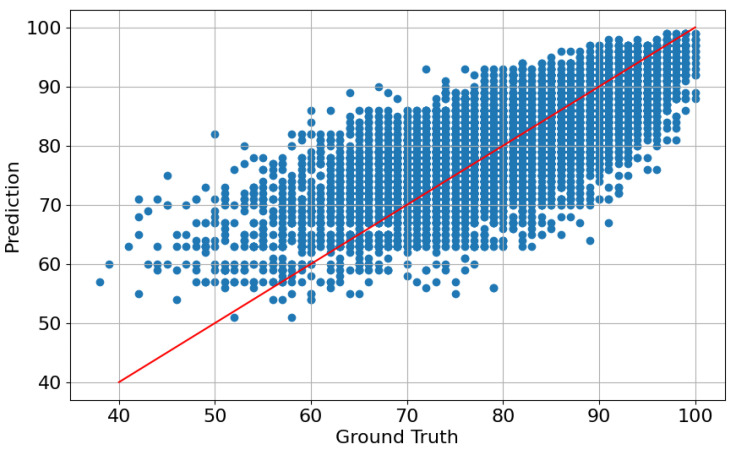
Scatter plot of the validation predictions and ground truths for the best-performing model on the five folds (r=128,α=1024). Each blue dot represents a video sample. The red line represents the perfect estimator: Ground Truth = Prediction.

**Figure 6:**
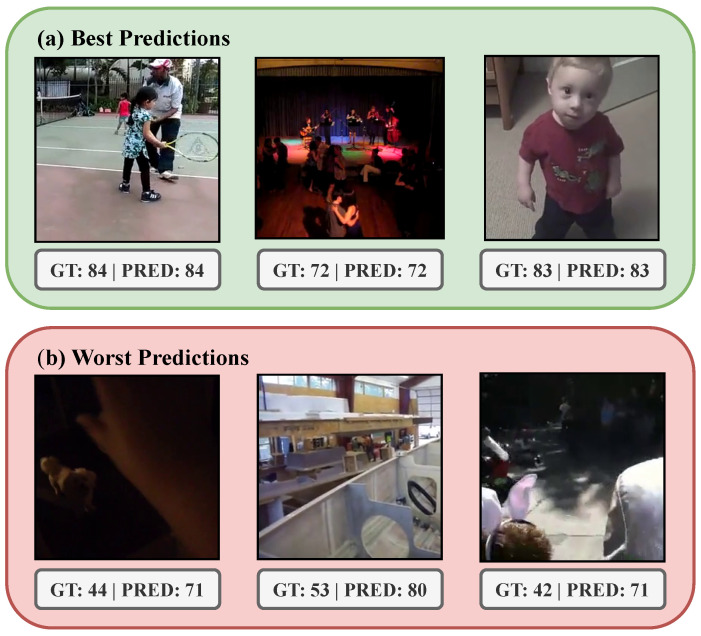
Best (**a**) and worst (**b**) memorability predicted examples from the validation set. GT: Ground Truth, PRED: Prediction.

Next, we explore the predictions obtained using the best model (r=128,α=1024). Figure 5 shows a scatter plot for the predicted and ground truth scores obtained using this checkpoint. It can be seen that, for most of the range, there is a clear correlation between both, in line with the obtained metric. However, it can also be noticed that the model tends to predict better samples with higher memorability, whilst it tends to assign higher values to the bottom-tier videos. In particular, there are no predicted scores below 50, whilst the ground truth values span below 40. This aspect might be derived from the fact that there are relatively few low-memorability examples in the dataset, which prevents the model from learning generalizable characteristics of videos that are associated with a lower probability of being remembered, or even including these lower values in its range of predictions. Moreover, as can be seen in Figure 6, the worst predicted examples are also in the low-memorability zone, supporting the hypothesis that the model lacks understanding of what makes a video forgettable. Visual inspection shows that the videos that are best predicted often show distinct objects or people performing clear actions such as playing, singing, or facing the camera in close-up shots. On the other hand, the worst-performing samples (which also tend to be lower-valued) often depict poorly lit scenarios or lack of a distinctive protagonist figure, let it be an object, animal, or person. This underperformance can be attributed to fundamental challenges in visual processing that directly impact memorability. Poor lighting reduces available visual information, leading to a lower signal-to-noise ratio, loss of detail, and color distortion. These degradations hinder the effectiveness of feature extraction by image encoders such as ViTs, creating a domain shift from well-lit training data. Furthermore, the absence of a distinct protagonist introduces ambiguity, making object and action recognition, as well as general scene understanding, more difficult. This lack of a clear focal point disrupts attention mechanisms, leading to less informative representations. Critically, these visual deficiencies directly impact memorability, as poorly lit and ambiguous scenes lacking a salient focus are inherently less memorable. This aligns with human perception research, which emphasizes the role of lighting, object salience, and scene complexity in memory encoding.

### 4.4. Comparison Against the State of the Art

After choosing the optimum hyperparameters, we train a new checkpoint with all available labeled data and perform inference on the unlabeled test samples, submitting a run to the MediaEval challenge organizers for a fair test evaluation on unseen data. Our system achieves an SRCC of **0.7444**, which notably surpasses previous peer-reviewed works under the same setup (see Table 1). This serves as evidence of the robustness of our method that even exceeds human consistency. It also highlights the adequate choice of experimental setup. By performing a Cross-Validation hyperparameter selection instead of evaluating over a single validation partition, we improve the robustness of our estimation of system performance. This is evidenced by a drop in performance from the validation results to the test results of 1.46%, which is relatively low for this problem and configuration. Previous state-of-the-art solutions relied on incorporating several visual and textual features through specialist modules [18,35] or training large vision backbones with a high amount of visual data per sample [31,37]. On the other hand, our approach leverages both textual and visual pre-training knowledge through PEFT and low-resource adaptation, using only three frames and five textual descriptions per video. In this way, we put this large-scale knowledge into service as a powerful video memorability prediction. Moreover, the QLoRA method enables this LVLM to be adapted for a downstream task with proficient results by training a limited fraction of the total parameters. For the best-performing configuration (r=128α=1024), out of the total 9.6 B model weights, only 67.1 M (0.7%) are updated. Although the original Qwen-VL model has a greater total number of parameters in comparison with previous state-of-the-art models, these strategies rely on either training from scratch or fine-tuning the whole network, failing to effectively leverage base knowledge acquired by foundation models. Our approach excels in applying minimal transformations to the base checkpoints in an effective manner, allowing model size to be scaled without increasing computational requirements by the same order of magnitude. Together with quantization, low-rank adaptation enables both training and inference of this otherwise expensive model on a single GPU.

### 4.5. Limitations

While our method demonstrates strong performance on the official test portion of Memento10k, the reference dataset in academic memorability prediction, several limitations remain. Memento10k is a highly specific dataset, featuring short, low-quality videos that typically depict a single semantic unit. This constrained setting raises questions about the generalizability to broader video types, such as movie excerpts, commercial advertisements, or educational multimedia. In such cases, the assumption that three frames can sufficiently represent an entire video may not hold, necessitating the use of LVLMs capable of processing full video sequences. Future work should explore whether the QLoRA adaptation strategy generalizes to such models and scenarios.

Additionally, while QLoRA fine-tuning enables efficient adaptation, our approach leverages a large-scale LVLM, Qwen-VL, which has a significantly higher parameter count than previous state-of-the-art models. Further research should investigate alternative video-processing LVLMs with comparable or smaller total parameter counts, balancing efficiency and performance.

## 5. Conclusions and Future Work

This study demonstrates the efficacy of adapting Large Vision–Language Models (LVLMs) for video memorability prediction, a task that takes advantage of the intrinsic relationship between semantic understanding and human perception. Using the QLoRA framework for parameter-efficient fine-tuning, we successfully transformed the Qwen-VL model into a state-of-the-art memorability predictor, achieving a Spearman Rank Correlation Coefficient (SRCC) of 0.744 on the Memento10k official test partition dataset. This significant improvement over existing methods underscores the potential to leverage generalist multimodal pre-training in specialized domains. Notably, our results demonstrate that although parameter-efficient methods maintain a significant portion of the underlying knowledge of the pre-trained model, they exhibit considerable sensitivity to task-specific adjustments.

Future work will focus on addressing the aforementioned limitations and expanding the scope of research in several directions. First, we aim to incorporate additional datasets to diversify the range of content and enhance the generalizability of the model. Second, alternative parameter-efficient adaptation methods, such as prompt-tuning and adapter-based learning, could be explored to further optimize performance. Third, integrating more sophisticated semantic representations, such as contextual embeddings from natural language descriptions or affective elements, may provide deeper insights into the cognitive mechanisms underlying memorability. The assimilation of additional modalities and more nuanced information is motivated by recent advances in rich, semantical audio–visual processing and video and scene understanding through efficient memory handling on LLMs, amongst others [66,67,68]. Finally, an investigation of fairness and bias in model predictions will be critical to ensure equitable outcomes in various types of content and audiences.

## Figures and Tables

**Table 1:** Comparison against the state of the art for our proposed solution. The results shown are reported over the official test partition of the Memento10k dataset. The number of parameters for all the approaches except ours are estimated from the available information in the published papers and related code releases. For Kumar et al. [37], the available information lacks fundamental structural details (i.e., number of layers and heads of the Transformer model) to provide a reasonable estimation. The best result in terms of SRCC is highlighted in bold. N/A stands for Not Available.

Model	Number of Total Parameters	Number of Trained Parameters (% Total)	SRCC (95% CI)
Human Consistency [18]	-	-	0.730
SemanticMemNet [18]	13 M	13 M (100%)	0.663 (0.634, 0.690)
Constantin, Ionescu [31]	57.7 M	57.7 M (100%)	0.665 (0.636, 0.692)
M3-S [35]	110 M	110 M (100%)	0.670 (0.641, 0.697)
Kumar et al. ^1^ [37]	N/A	N/A	0.713 (0.687, 0.737)
**Qwen-VL (QLoRA) (Ours)**	9.6 B	67.1 M (0.7%)	**0.744 (0.721, 0.766)**

^1^ Preprint, accepted for publication at WACV 2025.

## Data Availability

Restrictions apply to the availability of these data. Data were obtained from Newman et al. [18] and are available from the authors with their permission.
